# Quality of life of older people in nursing homes in China–evaluation and application of the Chinese version of the life satisfaction questionnaire

**DOI:** 10.1186/s12877-022-03040-4

**Published:** 2022-04-15

**Authors:** Yan Lou, Lijuan Xu, Marianne Carlsson, Xuefen Lan, Maria Engström

**Affiliations:** 1 Medicine College, Lishui University, No. 1 Xueyuan Road, Lishui City, China; 2 Department of Caring Science, Faculty of Health and Occupational Studies, University of Gävle, 801 76 Gävle, Sweden; 3 Department of Public Health and Caring Sciences, Uppsala University, Uppsala, Sweden

**Keywords:** Psychometric testing, Life satisfaction questionnaire, Quality of life, Older adults, Residential living homes, Influencing factors

## Abstract

**Background:**

Chinese and global populations are aging, and more older people are living in nursing homes in China. However, there is a lack of research measuring nursing home residents’ quality of life (QOL), and especially associations with nursing home types (publicly versus privately run). Therefore, this study aimed to determine the construct validity and internal consistency of the Chinese version of the life satisfaction questionnaire (LSQ-Chinese) and determine the associations between nursing home types (publicly versus privately run), residents’ sociodemographic characteristics, and their QOL.

**Methods:**

A cross-sectional survey measuring QOL among older people living in nursing homes was conducted (*n* = 419). Confirmatory factor analysis and Cronbach’s alpha were used to assess the construct validity and reliability of the LSQ-Chinese. In addition, multivariate regression analysis was used to examine these associations.

**Results:**

Confirmatory factor analysis indicated acceptable goodness-of-fit statistics for the seven-factor LSQ solution. All factors and the total scale had good internal consistency, with Cronbach’s alpha values > 0.70. The two factors with the highest QOL scores (higher scores indicate a more desirable state) were “physical symptoms” and “socioeconomic situation,” and those with the lowest QOL scores were “quality of close-friend relationships” and “quality of daily activities fun”. Residents living in privately run nursing homes had higher LSQ scores overall and for all factors except “physical symptoms” and “sickness impact” compared with publicly run nursing homes. Multivariate analyses indicated that marital status, number of chronic diseases, education level, main source of income, and nursing home type significantly contributed to the variance in the total LSQ scores. The associated sociodemographic variables differed between the factors, and the variable publicly versus privately run was significant for five of the seven factors.

**Conclusions:**

The LSQ is a suitable instrument for measuring the QOL of Chinese nursing home residents. The total LSQ score was higher among residents in privately run nursing homes than in publicly run ones. According to residents’ needs, staff should work for person-centered activities, and facilitate residents’ social interactions with friends, as both these aspects were scored relatively low.

**Supplementary Information:**

The online version contains supplementary material available at 10.1186/s12877-022-03040-4.

## Introduction

The age of the global population is increasing rapidly, and the proportion of people older than 60 years is estimated to reach 22% by 2050 [1]. In China, people aged 60 years or older already accounted for 19% of the total population in 2020 [2]. As the population has aged and the family situation has changed in China [3], the number of older people residing in nursing homes has increased [4]. There has also been a change from publicly run to government-constructed privately run nursing homes [5]. One important indicator of care quality in nursing homes is residents’ quality of life (QOL). However, there is sparse study on associations between nursing home types (publicly versus privately run) and resident-reported QOL. The present study aimed to psychometrically test a QOL instrument on older people living in nursing homes and determine associations between nursing home types (publicly versus privately run), residents’ sociodemographic characteristics, and their QOL.

## Background

According to several reviews, the instruments most frequently used to measure QOL among nursing home residents include the 36-item short form survey (SF-36), European quality of life five dimension questionnaire (EQ-5D), World Health Organization (WHO)QOL-BREF, and WHOQOL-OLD [6, 7]. These instruments are multidimensional [8–11] but differ in their contents. The EQ-5D and SF-36 are common health-related (HR) QOL instruments. These HRQOL instruments focus on health issues and limitations caused by health problems, for example, whether an individual can perform their usual activities [9, 11], but not the residents’ satisfaction with the activities itself. The WHOQOL-OLD is an instrument developed specifically for older people and suggested to be used together with the WHOQOL-BREF [8]. The WHOQOL-OLD includes the domains “sensory abilities,” “autonomy,” “past, present, and future activities,” “social participation,” “death and dying,” and “intimacy.” However, WHOQOL-OLD, if used alone, focuses less on perceived health, except for sensory abilities and activities partly in the past [8]. Regarding WHOQOL-OLD’s domain “death and dying,” the topic is sensitive to talk about in China [12, 13] and is not the main focus of the present study. Another interesting instrument is the life satisfaction questionnaire (LSQ) [14]. The strength of the LSQ is that it covers both perceived health and satisfaction with life situations, such as relationships, social situations, including living situations at the nursing home, and daily activities. One limitation is that the LSQ was initially developed to measure QOL in women with cancer [14]. However, it has subsequently been used among the general population [14, 15], older people living in nursing homes [16], and in the community [17]. Based on its domain, we considered the LSQ a suitable instrument for evaluating QOL among older people in nursing homes.

Influencing factors for QOL among nursing home residents found in previous studies include age [18, 19], sex [19–22] marital status [18, 21], education level [18, 19, 22, 23], economic situation [7, 18], nursing home ownership type [18], length of stay in a nursing home [21, 22], family member visits [18, 21], and multimorbidity [19, 21]. However, these relationships have also been found to differ based on the instrument used [24], included QOL domains and between different studies [18–22, 25], and are mostly examined in relation to QOL domains such as physical [18–22], psychological [18, 19, 21, 23], and some social health [2, 19] and function [25].

Nursing home ownership type has been indicated as an influencing factor for QOL, with higher scores for publicly run nursing homes [18, 26]. Regarding nursing home ownership type and care quality, a systematic review found that the four most common measures were staff quality, the prevalence of pressure ulcers, physical restraints, and governmental regulatory assessment deficiencies [27]. These quality outcomes are clearly of importance, as are the self-reported outcomes of residents, such as their QOL. The nursing home care system in China has recently undergone a transformation. Several publicly run nursing homes have changed into government-constructed privately run nursing homes to optimize the allocation of older adults’ care resources [5]. These nursing homes are owned by the general public and operate based on contracts with the private sector. The local government in the region where this study was performed selected publicly run nursing homes in urban areas in 2016 and asked several firms with experience in operating nursing homes to run them [28]. Thus, in addition to the current studies [18, 27], we were interested in examining the relationship between nursing home type (publicly versus privately run) and resident-reported QOL using an instrument covering both perceived health and residents’ satisfaction with life situations in the nursing home. Furthermore, we also wanted to evaluate sociodemographic variables, as these have been found to differ between different instruments, QOL domains, and studies, and are less explored in relation to QOL domains, such as satisfaction with daily activities in the nursing home and social relations.

The present study aimed to determine the construct validity and internal consistency of the Chinese version of the LSQ (LSQ-Chinese) and determine the associations between nursing home types (publicly versus privately run), residents’ sociodemographic characteristics, and their quality of life.

## Methods

### Setting and sample

All residents from 10 nursing homes in one city in southeast China were enrolled (six privately run and four government-run nursing homes [hereafter referred to as publicly run nursing homes]). Among the six privately run nursing homes, five were constructed by the government, and one was a private nursing home. All six privately run nursing homes were non-profit. These nursing homes provide daily life care, rehabilitation care, health care, spiritual comfort (spiritual comfort services, including communication and organized activities), psychological consultation, crisis intervention, and hospice care. Regarding the services provided, in addition to single rooms/apartments, double rooms/apartments, or mixed dormitories, nursing homes also have medical facilities, cultural activity rooms, and outdoor activity areas. The rooms in publicly run nursing homes were mostly shared by two residents, with separate rooms for males and females, except for a few rooms shared by married couples. The bathroom facilities were connected to the rooms, but there were no private kitchens. For privately run nursing homes, residents can choose to live alone or share an apartment with others. The apartments had bathroom facilities, and some of the privately run nursing homes also had kitchen facilities. In addition, all nursing homes had a café where food was provided. Nursing homes were staffed by frontline workers, including registered nurses, nursing aides, and administrative staff.

The inclusion criteria for participants were 60 years of age or older, being able to respond to the questionnaire in a structured interview, and having been provided informed consent. The response rate of the 460 distributed questionnaires was 95% (*n* = 437), and after removing questionnaires with missing LSQ data, 91% of the participants remained (*n* = 419). The mean age of the participants was 78.9 years (standard deviation [SD] = 9.6), slightly more than half were female (54.7%), and the median length of stay at the nursing home was 19 months. Table 1 presents the participant characteristics.

**Table 1:** Sociodemographic characteristics of participants

Variables	Values
Age mean (SD), years	78.9 (9.6), min 60 max100
Months residing at the nursing home Md (Q1;Q3)	19 (10;48)
Number of chronic diseases, n (%)	
≤1	328 (78.3)
≥2	83 (19.8)
Sex, n (%)	
Male	188 (44.9)
Female	229 (54.7)
Marital status, n (%)	
Divorced/single/widow(er)	291 (69.5)
Married	111 (26.5)
Education, n (%)	
No formal/primary school	252 (60.1)
Junior high school or higher	163 (38.9)
Main source of income, n (%)	
Retirement pension	203 (48.4)
Social assistance/family/others	211 (50.4)
MI, n (%)	
Basic for urban and rural residents or self-payed	226 (53.9)
For employees	188 (44.9)
Type of nursing home, n (%)	
Publicly run	135 (32.2)
Privately run	284 (67.8)

*Abbreviations*: *SD* Standard deviation, *Md* Median, *Q* Quartile, *MI* Medical insurance, When sum does not add up to *n* = 419 or 100% there are internal missing data

### Data collection

Data were collected from July 2017 to April 2018 after obtaining permission from nursing home managers. In addition, the data collectors were provided with sufficient training to ensure consistent results.

### Questionnaire

QOL was measured using the LSQ. The LSQ contains 34 items that cover the following factors: “physical symptoms” (PS) seven items, “sickness impact” (SI) six items, “quality of daily activities fun” (QDAF) three items, “quality of daily activities meaningful” (QDAM) four items, “socioeconomic situation” (SES) three items, “quality of family relation” (QFR) five items and “quality of close-friend relationship” (QCFR) five items and one single measurement/item of overall QOL [15]. The response alternatives are 7-grade. For total and factor scores, items are summarized, divided by the highest possible score, and multiplied by 100. The possible QOL scores range from 14 to 100, with 100 representing the most desired value. The developer of the original LSQ (third author) was one of the co-authors of the present study. The LSQ is not licensed and is free to use. The sociodemographic characteristics measured were age, sex, length of stay at the nursing home, marital status, education level, main source of income, number of chronic diseases, and medical insurance types.

Two bilingual nursing teachers independently translated the LSQ into Chinese (Mandarin) and discussed it with a third person to reach a consensus for the translation. Back-translation was performed independently on the LSQ by two bilingual-speaking experts to achieve semantic equivalence [29]. This back-translated version was compared with the original version, all co-authors (China and Sweden) discussed it, and minor adjustments were made, such as items “quality of relations – fun/stimulating” being changed to “quality of relations – interesting/exciting,” and “quality of relations – varied” being changed to “quality of relations – changeable.”

### Data analysis

Data were analyzed using IBM SPSS Statistics (version 24) and IBM SPSS Amos. Descriptive data are presented as means and SD, numbers, and percentages. The groups were compared using the Student’s t-test, and bivariate and multivariate relationships were determined using Pearson’s correlation coefficient (r) and multivariate linear regression. Visual inspection of the regression standardized residuals, histograms, and normal q-q plots revealed no serious deviations from a normal distribution. Among the included independent variables, multicollinearity was assessed using the variance inflation factor (all values ≤1.414 in the multivariate analyses), with a value less than 2.5 considered an acceptable value [30]. The variables of the number of diseases, education level, and length of stay at the nursing home were dichotomized based on the group median. Confirmatory factor analysis (CFA) were performed using Amos with the maximum likelihood estimation method to determine the construct validity of the LSQ. Using the results from the modification indices, we allowed some error terms that were within the same factor or judged as reasonable to correlate. Goodness-of-fit statistics for the different models are presented using absolute parameters, such as chi-square/degrees of freedom (χ^2^/df) (< 3) [31] and root-mean-square error of approximation (RMSEA) with 90% confidence intervals, with acceptable values < 0.08 [32], and the upper bound does not exceed 0.10 [33]. Furthermore, we used relative parameters, such as the comparative fit index (CFI), where good values are > 0.95, but > 0.90, are also acceptable [31]. Finally, internal consistency for the factors and the total LSQ scale was measured using Cronbach’s alpha values. The criterion for statistical significance was set at *P* < 0.05.

### Ethical considerations

This study was approved by the medical ethics committee of the university to which the first, second, and fourth authors are affiliated (no. 2017–0211). For each nursing home, residents were first assembled in one room to receive oral information regarding the study. Those interested in participation could tell the data collectors directly or the staff after the meeting. All participants also received oral and written information before data collection and signed an informed consent form. In the written and oral information, it was clear that participation was strictly voluntary, would not affect their care and social services in any way, and that they could end participation at any time during the study. Soaps and towels were offered as gifts to participants. Residents were assured that their responses would remain confidential, and the researchers and participants were not related.

## Results

### Construct validity and internal consistency of the LSQ

**Fig. 1:**
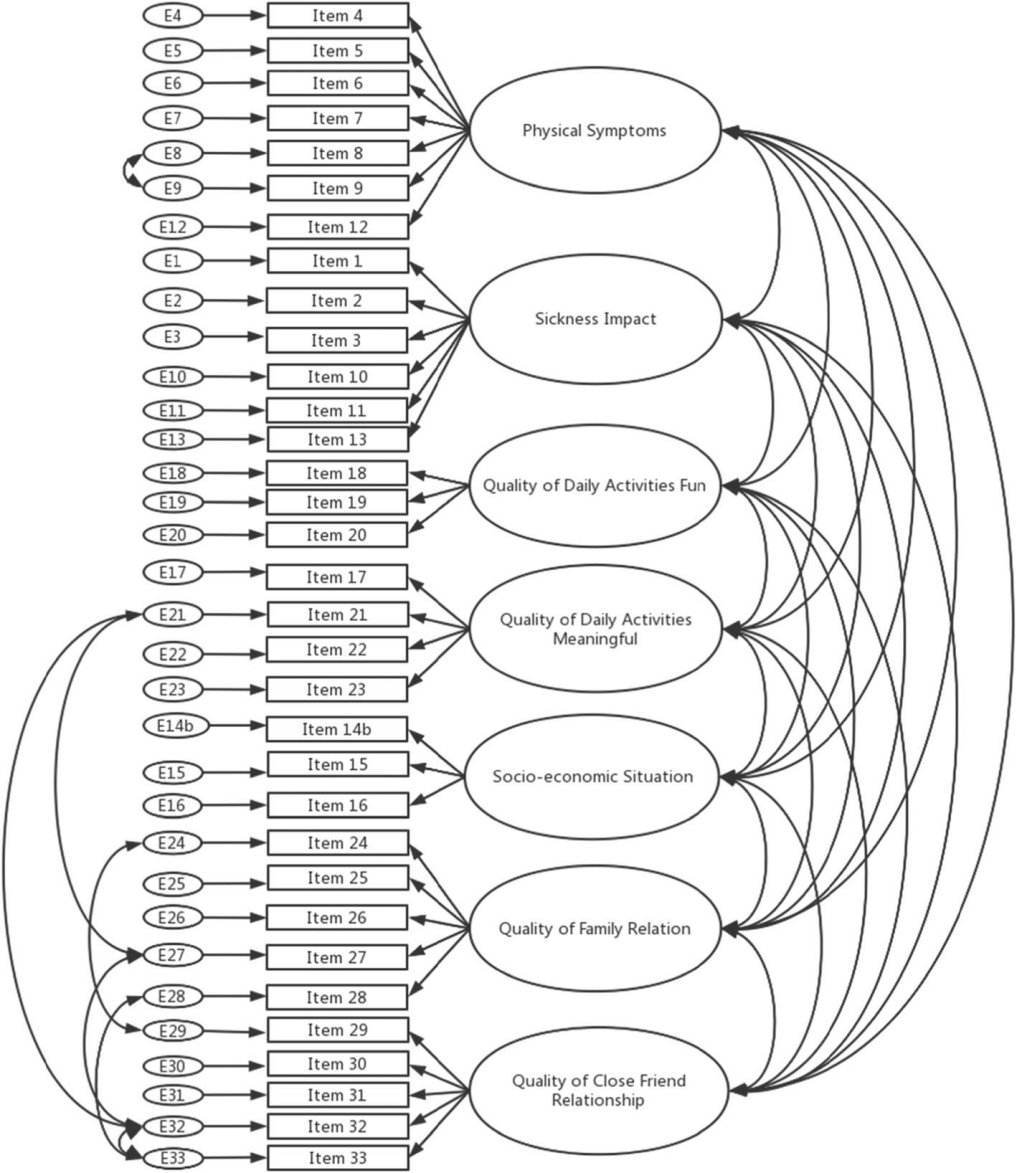
Flow chart presenting the underlying construct of the Life satisfaction Questionnaire Chinese version ([error term] E)

Based on the recommendations of the Swedish LSQ [15], we started with a seven-factor solution; however, we also tested a six-factor solution that has been presented as an alternative. Goodness-of-fit statistics for Model 1a (allowing the seven factors to correlate and some error terms based on modification indices) were acceptable/within recommended values (χ^2^/df = 2.988, CFI = 0.901, and RMSEA = 0.069), while Model 2 (six-factor solution) had χ^2^/df > 3 and CFI < 0.9 (χ^2^/df = 3.228, CFI = 0.890, and RMSEA = 0.073). Table 2 lists the goodness-of-fit statistics for Models 1 and 2, and Fig. 1 shows the items and factors in Model 1. We also tested the second-order structure of Model 1b, as presented in the Swedish publication, CFA of LSQ [15], adding the second-order factors of physical functioning (based on PS and SI), daily living (QDAF, QDAM, and SES), and personal relationships (QFR and QCFR). The goodness-of-fit statistics for this model did not improve compared with Model 1a (χ^2^/df = 3.198, CFI = 0.887, and RMSEA = 0.073).

**Table 2:** Goodness of fit values for Model 1 and 2, standardized regression weights for the items in Model 1 and 2 and descriptive data for the items in Model 1

	**Model fit summary**			
	**Model 1**		**Model 2**	
Chi-square (df) *p*-value	1395.6 (467) < 0.001		1484.9 (460) < 0.001	
Chi-square/df	2.99		3.23	
CFI	0.901		0.890	
RMSEA (90 CIs)	0.069 (0.065;0.073)		0.073 (0.069; 0.077)	
Items	**Factor loading**		**Mean (SD)**	**Median (Q1;Q3)**
	**Model 1**	**Model 2**	**Model 1**	
1. Tiredness	0.764	0.739	4.6 (1.7)	5 (3;6)
2. Lack of fitness	0.815	0.787	4.7 (1.7)	5 (3;6)
3. Sleep disturbances	0.507	0.484	4.4 (1.9)	4 (3;6)
4. Loss of appetite	0.553	0.543	4.8 (1.8)	5 (4;7)
5. Diarrhoea	0.618	0.600	5.7 (1.6)	6 (5;7)
6. Constipation	0.630	0.608	5.2 (1.8)	5 (4;7)
7. Dizziness	0.668	0.676	4.8 (1.8)	5 (3;7)
8. Palpitation of the heart	0.719	0.700	5.3 (1.6)	5 (4;7)
9. Breathing difficulties	0.696	0.702	5.5 (1.6)	6 (4;7)
10. Muscular weakness	0.765	0.767	4.7 (1.8)	5 (3;6)
11. Pain	0.697	0.725	4.6 (1.7)	4 (3;6)
12. Nausea	0.708	0.737	5.6 (1.5)	6 (4;7)
13. How do you perceive overall health?	0.637	0.646	4.4 (1.3)	4 (4;5)
14. If you are not gainfully employed, how happy are you with your life situation?	0.856	0.853	5.0 (1.3)	5 (4;6)
15. Describe your financial situation	0.751	0.753	4.8 (1.3)	4 (4;6)
16. Are you happy with where you live?	0.773	0.773	5.2 (1.3)	5 (4;6)
17. How active have you been during the past week?	0.658	0.676	4.5 (1.2)	4 (4;5)
How do you feel about your activities (i.e. what you have done) during the past week? To what extent have your activities been: (18-23)				
18. Fun/stimulating	0.919	0.813	4.4 (1.6)	4 (3;6)
19. Interesting	0.970	0.881	4.3 (1.6)	4 (3;5)
20. Creative	0.615	0.636	3.4 (1.7)	3 (2;5)
21. Independent	0.588	0.556	4.4 (1.7)	5 (3;6)
22. Useful	0.856	0.826	4.3 (1.6)	4 (3;5)
23. Meaningful	0.811	0.779	3.9 (1.8)	4 (3;5)
How do you experience your relationships with other people? Choose and assess a significant person from your family, to what extent do you feel the relationship is:				
24. Emotionally satisfying	0.899	0.902	4.8 (1.8)	5 (4;6)
25. Interesting/exciting	0.913	0.914	4.7 (1.8)	5 (4;6)
26. Meaningful	0.942	0.941	4.5 (1.8)	5 (3;6)
27. Independent	0.383	0.369	4.3 (1.8)	5 (3;6)
28. Changeable	0.177	0.188	3.0 (1.8)	3 (1;4)
Choose and assess a significant person from among your friends. To what extent do you feel the relationship is:				
29. Emotionally satisfying	0.892	0.890	4.4 (1.7)	5 (3;6)
30. Interesting/exciting	0.941	0.941	4.3 (1.8)	4 (3;6)
31. Meaningful	0.951	0.949	4.3 (1.7)	4 (3;5)
32. Independent	0.547	0.526	4.1 (1.8)	4 (3;5)
33. Changeable	0.282	0.279	2.8 (1.7)	3 (1;4)

*Abbreviations*: *df* Degrees of freedom, *CFI* Comparative Fit Index, *RMSEA* Root Mean Square Error of Approximation, *CIs* Confidence Intervals, *SD* Standard Deviation, *Q* Quartile

Three items in Models 1a and 2 had factor loadings below 0.5: items 27 (“family relations – independent”), 28 (“family relations – changeable”), and 33 (“close-friend relations – changeable”). Item 3 (“sleep disturbances’) in Model 2 also had a factor loading below 0.5 (Table 2). The Cronbach’s alpha of internal consistency for Model 1a ranged from 0.77 (QFR) to 0.86 (QDAF) for the factors and was 0.93 for the total LSQ (Table 3).

**Table 3:** Quality-of-life compared for different subgroups

	PS	SI	QDAF	QDAM	SES	QFR	QCFR	LSQ
Mean (SD), all participants	75.3 (17.0)	65.5 (18.1)	57.2 (20.9)	61.4 (18.1)	71.2 (16.2)	60.9 (18.4)	57.1 (19.7)	64.9 (13.5)
Sex, *p*-value^a^	**< 0.001**	**0.001**	**0.005**	**0.006**	0.785	0.121	0.430	0.511
Male	**79.4 (16.0)**	**68.6 (19.0)**	54.0 (21.6)	58.7 (18.2)	71.4 (17.3)	59.3 (19.6)	56.2 (19.4)	65.3 (13.7)
Female	72.0 (17.1)	62.9 (17.0)	**59.8 (20.1)**	**63.5 (17.7)**	71.0 (15.4)	62.1 (17.4)	57.8 (19.9)	64.5 (13.4)
Marital status, *p*-value^a^	**< 0.001**	**< 0.001**	**< 0.001**	**< 0.001**	**< 0.001**	**< 0.001**	**< 0.001**	**< 0.001**
Divorced/single/widower	72.5 (16.5)	62.7 (17.3)	53.2 (20.2)	58.0 (17.3)	68.3 (16.2)	57.4 (19.6)	54.0 (19.6)	61.7 (12.8)
Married	**82.2 (15.4)**	**72.1 (17.5)**	**66.2 (19.8)**	**69.5 (17.2)**	76.6 (13.6)	68.1 (11.4)	62.9 (17.1)	**71.8 (11.5)**
Chronic Disease, *p*-value^a^	**0.002**	**0.005**	**0.029**	**0.021**	0.101	0.824	0.190	**0.005**
0-1	**77.0 (16.1)**	**67.0 (17.8)**	**58.4 (20.6)**	**62.6 (17.8)**	72.0 (15.7)	61.2 (18.4)	58.0 (18.8)	**66.0 (13.1)**
2 or more	70.0 (18.9)	60.9 (18.3)	52.7 (21.9)	57.5 (19.0)	68.7 (18.4)	60.6 (18.6)	54.4 (22.5)	61.4 (14.7)
Education, *p*-value^a^	**< 0.001**	**< 0.001**	**< 0.001**	**< 0.001**	**< 0.001**	**< 0.001**	**< 0.001**	**< 0.001**
No formal/primary school	72.4 (16.5)	62.3 (17.7)	53.2 (20.9)	58.1 (17.6)	69.0 (15.9)	56.3 (19.3)	52.8 (18.8)	61.4 (13.0)
Junior high school or more	**80.4 (16.6)**	**70.8 (17.5)**	**63.6 (19.6)**	**66.7 (17.8)**	**74.7 (16.2)**	**67.9 (14.7)**	**63.4 (19.6)**	**70.5 (12.5)**
Medical insurance, *p*-value^a^	**< 0.001**	**< 0.001**	**< 0.001**	**< 0.001**	**< 0.001**	**< 0.001**	**< 0.001**	**< 0.001**
Basic for rural and urban residents or self-payed	71.6 (16.3)	62.2 (16.9)	52.9 (21.0)	57.3 (18.2)	66.8 (16.2)	57.0 (19.0)	52.4 (20.3)	60.9 (13.1)
For employees	**80.4 (16.5)**	**69.9 (18.6)**	**62.3 (19.9)**	**66.4 (16.8)**	**76.5 (14.7)**	**65.4 (16.9)**	**62.4 (17.7)**	**69.8 (12.5)**
Main source income, *p*-value^a^	**< 0.001**	**< 0.001**	**< 0.001**	**< 0.001**	**< 0.001**	**0.031**	**< 0.001**	**< 0.001**
Retirement pension	**80.3 (16.0)**	**69.6 (17.8)**	**60.8 (18.9)**	**65.5 (16.1)**	**77.4 (13.6)**	**62.8 (18.0)**	**61.3 (17.3)**	**69.0 (11.8)**
Family, Social assistant/other	71.0 (16.6)	62.0 (17.5)	53.7 (22.4)	57.5 (19.2)	65.4 (16.5)	58.9 (18.9)	52.8 (21.1)	61.0 (14.1)
Residing at nursing home, *p*-value^a^	0.051	0.180	0.292	0.078	**0.004**	0.317	0.530	0.125
19 months or less	74.0 (16.4)	64.6 (17.5)	56.4 (22.4)	60.3 (19.0)	69.3 (16.6)	62.2 (18.5)	56.9 (20.9)	64.2 (13.8)
20 months or more	77.2 (16.7)	67.0 (18.0)	58.6 (19.2)	63.4 (16.8)	**73.9 (15.3)**	60.3 (17.8)	58.1 (17.9)	66.2 (12.6)
Nursing home, *p*-value^a^	0.089	0.094	**< 0.001**	**< 0.001**	**< 0.001**	**< 0.001**	**< 0.001**	**< 0.001**
Publicly run	73.3 (16.8)	63.3 (18.5)	45.4 (18.8)	53.2 (16.7)	66.0 (18.4)	48.2 (19.0)	47.1 (16.9)	58.1 (13.2)
Privately run	76.3 (17.0)	66.5 (17.9)	**62.8 (19.5)**	**65.3 (17.5)**	**73.7 (14.5)**	**66.9 (14.8)**	**61.9 (19.2)**	**68.1 (12.4)**
Cronbach’s Alpha values	0.84	0.84	0.86	0.81	0.84	0.77	0.84	0.93

*Abbreviations*: *LSQ* Life satisfaction questionnaire, *PS* Physical symptoms, (*SI* Sickness impact, *QDAF* Quality of daily activities fun, *QDAM* Quality of daily activities meaningful, *SES* Socio-economic situation, *QFR* Quality of family relation, and *QCFR* Quality of close friend relationship, *SD* Standard deviation

^a^*p*-value for student t-test. For the variable Education, Chronic diseases and Residing at the nursing home the data were dichotomized based on the group median value. For the LSQ and included factors the scores range from 14 to 100, with 100 representing the most desired value. Bold text for *p*-value indicate statistically significant values

### QOL among older people living in nursing homes

The three highest QOL scores (higher values correspond to a more desirable state) were observed for PS (mean = 75.3, SD = 17.0), SES (mean = 71.2, SD = 16.2), and SI (mean = 65.5, SD = 18.1), whereas the lowest scores were observed for QCFR (mean = 57.1, SD = 19.7), QDAF (mean = 57.2, SD = 20.9), and QFR (mean = 60.9, SD = 18.4) (Table 3). A comparison of scores for the factors within the group indicated statistically significant differences in the multivariate effect (Pillai’s trace F-statistic 80.516, hypothesis df 6, *P* < 0.001). Post-hoc pairwise comparisons revealed statistically significant differences between all factors (*P*-values ranged from < 0.001 to 0.002, with multiple comparison adjustments using the Bonferroni correction), except between QDAF and QCFR, and between QDAM and QFR.

### QOL, sociodemographic characteristics, and types of nursing home

Regarding sociodemographic characteristics, male had higher scores than female for the physical functioning factors PS and SI, whereas female had higher scores for the two factors of quality of daily activities, QDAF and QDAM. Participants who had one or no disease versus those with two or more diseases scored higher on the total LSQ and factors PS, SI, QDAF, and QDAM. Furthermore, higher scores were found for all factors and the total LSQ for married participants compared with divorced/single/widow(er), junior high school, or higher education level compared with no formal or primary school education; participants with medical insurance from employees compared with basic medical insurance for urban and rural residents or self-paid; and those with the main source of income from retirement pension compared with those from family/social assistance/other. Higher scores were also found for participants living in privately run nursing homes compared with publicly run nursing homes for the total LSQ and all factors, except PS and SI. Participants who had lived at a nursing home for ≥20 months had a higher SES score than those with shorter lengths of stay.

Table 4 presents the results of the multivariate regression analyses of the independent variables (sociodemographic characteristics and type of nursing home) and the outcomes of the total LSQ score and different factors. The different outcomes explained 13% (factor SI) and 25% (factor QFR and total LSQ score) of the variance in the results. The variables that contributed to significant proportions of the variance in the total LSQ score were marital status, number of chronic diseases, education level, main source of income, and type of nursing home. The significant variables for the physical functioning factors, PS and SI, were sex, marital status, number of chronic diseases, main source of income (only for PS), and education level (only for SI). The significant variables for quality of daily activities, both QDAF and QDAM, were sex, marital status, number of chronic diseases, and type of nursing home; for QDAM, the main source of income was also significant. The significant variables for the SES factor were marital status, main source of income, type of nursing home, and length of stay at the nursing home. The significant variables for quality of relations were QFR marital status, education level, main source of income, and type of nursing home, whereas for the QCFR type of nursing home.

**Table 4:** Multivariate linear regression analyses with LSQ total score and the different factors as dependent variables and personal characteristics as independent variables (significant variables^a^ from the analyses presented in Table 3), standardized regression coefficients

	LSQ tot	PS	SI	QDAF	QDAM	SES	QFR	QCFR
R square (adjusted)	0.25 (0.24)	0.18 (0.17)	0.13 (0.12)	0.21 (0.20)	0.19 (0.18)	0.18 (0.17)	0.25 (0.24)	0.14 (0.13)
Sex		− 0.177***	−0.133**	0.119*	0.146**			
Male (0)								
Female (1)								
Marital Status	0.219***	0.188***	0.163**	0.181***	0.195***	0.130*	0.132**	0.091 ns
Single/divorced/widow(er) (0)								
Married (1)								
Chronic disease(s)	−0.186***	− 0.180***	− 0.164**	−0.145**	− 0.156**			
None to one (0)								
Two or more (1)								
Education	0.121*	0.094 ns	0.142**	0.066 ns	0.068 ns	−0.087 ns	0.125*	0.077 ns
No formal/primary school (0)								
Junior high school or more (1)								
Main source of income^a^	−0.131**	−0.166**	− 0.088 ns	−0.046 ns	− 0.121*	−0.319***	0.098*	−0.079 ns
Retirement (0)								
Social assistant/from family/others (1)								
Nursing home	0.206***			0.275***	0.173**	0.155**	0.398***	0.264***
Publicly run (0)								
Privately run (1)								
Residing at the nursing home						0.113*		
19 months or less (0)								
20 months or more (1)								

For the variable Education, Chronic diseases and Residing at the nursing home the data were dichotomized based on the group median value

For the LSQ and included factors the scores range from 14 to 100, with 100 representing the most desired value. * *p* < 0.05,**p < 0.01, *** *p* < 0.001, ns = non-significant, Bold text in the table indicated statistically significant values. ^a^ When both main source of income and medical insurance were entered in the regressions models variance inflation factors indicated multicollinearity, and thereof only main source of income was included in the analyses

## Discussion

We evaluated the psychometric properties of the LSQ-Chinese and used a questionnaire to determine factors influencing QOL among older people in nursing homes. The results indicated that the LSQ-Chinese has good internal consistency and acceptable construct validity. Multivariate analysis indicated that being married, having a higher education level, and living in a privately run nursing home were positively correlated with total LSQ scores. Whereas, having two or more diseases and the main source of income from social assistance/family were negatively associated with total LSQ scores.

Regarding construct validity, the results of the CFA in the present study indicated that the seven-factor model had the best fit. Therefore, this model was chosen for our study and is recommended for future research and use in clinical practice among Chinese nursing home residents. When testing for construct validity, the quality of daily activities factor was split into QDAF and QDAM, as recommended by the authors of the original Swedish version of the LSQ, [15]. Item 34 (“evaluation of overall QOL”) was not included because each LSQ factor evaluates a portion of QOL, as recommended by Carlsson and Hamrin [15].

Items 27 (“family relations – independent”), 28 (‘family relations – changeable”), and 33 (“close-friend relations – changeable”) had low factor loadings [34]. As these three items were included in the Swedish LSQ and are important parts of the QFR and QCFR factors, we decided to include them for consistency with the Swedish LSQ. During data collection, some older adults said that “we are living in a nursing home, and now my life is not dependent on my family members but dependent on nursing home staff,” which may be a reason for the low factor loading of item 27 (“family relations – independent”). When answering items 28 and 33 about changeable relationships, the results suggested that the relationships varied to make things better or worse, which might have influenced the factor loadings. The earlier English translation of the Swedish LSQ that was also used to translate the LSQ-Chinese might have been misleading since Swedish items 28 and 33 stand for multifaceted/richness of variation. These will now be changed in the LSQ-Chinese to “family relations richness of variation” and “close-friend relations richness of variation.”

The highest score (indicating a more desirable state) among the QOL factors was for PS, with a mean of 75.3. The lowest scores were for activities QDAF and relationships QCFR, with mean scores of 57.2 and 57.1, respectively. These findings were similar to those of a Swedish study of nursing home residents and found that the highest and lowest scores were for PS and quality of activities, respectively [16]. During data collection, we learned that most nursing homes could provide residents with regulated rehabilitation and physical health care, while daily activities for spiritual comfort were limited to exercise in the morning and watching television in the afternoon, and some nursing homes had entertainment activities such as dancing, singing, or handwork (e.g., paper-folding). These results are consistent with a recent survey [35] of demands for nursing services among nursing home residents, in which residents reported the highest scores for their need for spiritual culture and entertainment (i.e., a need for more such activities), while they were more satisfied with medical care and rehabilitation. Our findings indicated that QOL domains such as quality of activities and close-friend relationships were poor among Chinese nursing home residents. Choi et al. [36] suggested that nursing home staff should provide different types of spiritual services and encourage older people to participate in entertainment and social activities to achieve positive health outcomes. The LSQ-Chinese version will be a valuable instrument to assess the quality of everyday activities in nursing homes and the quality of social relationships and perceived health. If used in clinical practice and in combination with discussions of what kind of activities each resident prefers, it could help staff offer more person-centered activities and stimulate social interaction.

Our results indicated that the type of nursing home (publicly versus privately run) was significantly associated with QOL. In privately run nursing homes, older people had higher scores for total QOL and all factors except PS and SI (Table 4). Non-significant results for physical and psychological domains were also found in a Chinese national study of people aged 60 years or older when comparing publicly run and government-constructed privately run nursing homes [18]. However, when they combined privately run and government-constructed privately run nursing homes in one group and compared them with the public run, the residents in public nursing homes had higher levels of QOL in the physical domain [18]. Another Chinese study [26] found that residents in publicly run nursing homes scored higher than those in private ones for total QOL and physical and mental health domains. Data were collected before the Chinese government started constructing privately run nursing homes. The authors considered that the higher QOL among residents was caused by higher medical staffing levels in publicly run nursing homes, and suggested that higher medical staffing levels provide better medical care for residents with acute diseases or health problems [26]. A Canadian study also indicated that residents aged 65 years or older in publicly run facilities had higher total QOL scores than those in privately run (for-profit or non-profit) or charitable facilities, and profit status was not related to the QOL of residents [37].

The inconsistencies between our results and one of the previous Chinese studies [26] might be related to timing and regional factors. During the transformation of the Chinese nursing home care system based on national recommendations, the government did not apply land tenancy fees to these firms [38]. Government-constructed privately run nursing homes usually employ different methods for modifying the care environment, such as ordering entertainment facilities and applying staff training to improve care quality using funds saved from not having to pay land tenancy fees. Most of the privately run nursing homes (five of six) in our study were government-constructed privately run nursing homes that were recently transformed from publicly run nursing homes. The transformation into government-constructed privately run nursing homes might have influenced our results since QOL levels were higher in privately run nursing homes than in publicly run ones.

Multivariate linear regression analysis indicated that the significant factors influencing QOL (total LSQ score) included marital status, number of chronic diseases, education level, main source of income, and nursing home type. In four of the seven factors of the LSQ, sex was a significant influencing factor according to multivariate analyses. Being male was positively associated with higher (i.e., better) scores in the physical function factors PS and SI but negatively associated with the daily activities factors QDAF and QDAM. Males having better physical function scores were consistent with most previous studies on nursing home residents [19–21]. Costa and Neri [39] suggested that one possible reason for females’ higher daily social activities was that females were more likely to participate in social activities, which is in accordance with our results of females having higher levels in QDAF and QDAM.

Marital status, number of chronic diseases, and educational level were also associated with QOL. These results were consistent with previous studies, where being married, without multimorbidity, and having higher education levels were positively associated with physical and psychosocial domains of QOL among older adults in nursing homes [18, 19, 23]. Our findings also indicated that being married and having higher education levels were positively associated with the quality of daily activities and relationships. In addition, the main source of income, being social assistance/from family/others, was negatively associated with QOL according to the multivariate analyses in our study, with a lower total LSQ score, PS, SES, and QDAM, but not QFR, which had a positive association. These results are consistent with previous studies. Older people who received a retirement pension had a higher overall QOL and better psychological well-being than those receiving income support from the government or family members [40, 41]. When meeting with the residents during data collection, we learned that most of those with the main income source from others received income from family members. This is a possible reason why this group of older people had higher QFR ratings in the present study.

### Methodological considerations

The strengths of the present study include high response rate and a few missing internal data points. These missing internal data were substituted with the median of the individuals for that factor. The used instrument, LSQ-Chinese had a high Cronbach’s alpha values for internal consistency, ranging from 0.77 to 0.86 for the factors and 0.93 for the total scale. CFA indicated acceptable construct validity. The study also has some limitations. One limitation was the cross-sectional design, which restricted the ability to conclude cause-effect relationships. Another limitation was the convenience sampling method, and data were collected from nursing homes in only one city in China and its surroundings, which could influence generalizability. However, participants’ sociodemographic characteristics were similar to those of other Chinese studies of older people’s needs in nursing homes and QOL using national data of nursing home residents [7, 36]. Additionally, for the included privately run nursing homes, we selected data from five government-constructed privately run nursing homes and one private nursing home. Future studies could compare the QOL of older people between the three different types of nursing home and should include their profit status. Regarding data collection, there may have been gatekeeper problems, as it was the staff who told the residents about the study’s information meeting. Regarding the inclusion criteria, “being able to respond to the questionnaire in a structured interview” was not tested with any tests for mental capacity or cognitive function assessment. The last limitation was that test-retest reliability was not determined for the LSQ-Chinese.

## Conclusions

Our findings provide evidence that the LSQ-Chinese has good reliability and acceptable validity and is suitable for assessing QOL among older people in Chinese nursing homes. Compared with residents in publicly run nursing homes, those in privately run nursing homes had better QOL scores in total QOL, and five of the seven QOL domains. Moreover, being married, having no to one disease versus two or more, having higher education levels, and being the main source of income from retirement versus social assistance/from family/others were associated with better total LSQ scores. In addition, the study found low scores among residents in the QOL domains of quality of daily activities and close-friend relationships. Future interventional studies are needed to explore measures for improving the quality of relationships and daily activities of residents to improve QOL and allow healthy aging among nursing home residents.

## Supplementary Information

**Additional file 1.** Survey about Quality of Life among Older People in Nursing Home.

## Data Availability

The dataset generated during the current study is available from the corresponding author on reasonable request.

## Abbreviations

CFA: Confirmatory factor analysis
CFI: Comparative Fit Index
EQ-5D: European Quality of Life Five Dimensions questionnaire
LSQ: Life Satisfaction Questionnaire
PS: Physical symptoms
QCFR: Quality of close friend relationship
QDAF: Quality of daily activities fun
QDAM: Quality of daily activities meaningful
QFR: Quality of family relation
QOL: Quality Of Life
RMSEA: Root Mean Square Error of Approximation
SES: Socio-economic situation
SD: Standard deviation
SF-36: Short Form Survey-36
SI: Sickness impact
WHO: World Health Organization
