# Supplementary material for: Quality of life of older people in nursing homes in China–evaluation and application of the Chinese version of the life satisfaction questionnaire
Source: BMC Geriatr. 2022 Apr 15;22:328. doi: 10.1186/s12877-022-03040-4 (PMC9013118; doi:10.1186/s12877-022-03040-4)
Supplement: Supplementary file 1 — Additional file 1. Survey about Quality of Life among Older People in Nursing Home. [file 12877_2022_3040_MOESM1_ESM.docx]

**Survey about Quality of Life among Older People in Nursing Home**

**Part one: Demographic characteristics of the participants**

1. Age: years
2. Gender： □Male □Female
3. Marital status：□Divorced □single □widower □Married

4. Months residing at the nursing home: .

5. Number of chronic diseases: .

6. Education：□No formal □Primary school □Junior high school

□Senior high school □College/University

7. Main source of income: □Retirement pension □From family/others

□Social assistant

8. Medical insurance: □Basic MI for urban and rural residents □MI self-payed

□MI for employees

**Part two:**

The questions below deal with your health and life situation. The information you give will be treated confidentially.

Please answer all the questions.Which of the symptoms or problems below have caused you difficulties during the past week? Put an X in the box that best correspond to your experience

**To what extent have you been troubled by:**

**1. Tiredness 2. Lack of fitness**

To a very high degree ( ) To a very high degree ( )

To a high degree ( ) To a high degree ( )

To a fairly high degree ( ) To a fairly high degree ( )

To some degree ( ) To some degree ( )

To a low degree ( ) To a low degree ( )

Almost not at all ( ) Almost not at all ( )

Not at all ( ) Not at all ( )

**3. Sleep disturbances 4. Loss of appetite**

To a very high degree ( ) To a very high degree ( )

To a high degree ( ) To a high degree ( )

To a fairly high degree ( ) To a fairly high degree ( )

To some degree ( ) To some degree ( )

To a low degree ( ) To a low degree ( )

Almost not at all ( ) Almost not at all ( )

Not at all ( ) Not at all ( )

**5. Diarrhoea 6. Constipation**

To a very high degree ( ) To a very high degree ( )

To a high degree ( ) To a high degree ( )

To a fairly high degree ( ) To a fairly high degree ( )

To some degree ( ) To some degree ( )

To a low degree ( ) To a low degree ( )

Almost not at all ( ) Almost not at all ( )

Not at all ( ) Not at all ( )

**7. Dizziness 8. Palpitation of the heart**

To a very high degree ( ) To a very high degree ( )

To a high degree ( ) To a high degree ( )

To a fairly high degree ( ) To a fairly high degree ( )

To some degree ( ) To some degree ( )

To a low degree ( ) To a low degree ( )

Almost not at all ( ) Almost not at all ( )

Not at all ( ) Not at all ( )

**9. Breathing difficulties 10. Muscular weakness**

To a very high degree ( ) To a very high degree ( )

To a high degree ( ) To a high degree ( )

To a fairly high degree ( ) To a fairly high degree ( )

To some degree ( ) To some degree ( )

To a low degree ( ) To a low degree ( )

Almost not at all ( ) Almost not at all ( )

Not at all ( ) Not at all ( )

**11. Pain 12. Nausea**

To a very high degree ( ) To a very high degree ( )

To a high degree ( ) To a high degree ( )

To a fairly high degree ( ) To a fairly high degree ( )

To some degree ( ) To some degree ( )

To a low degree ( ) To a low degree ( )

Almost not at all ( ) Almost not at all ( )

Not at all ( ) Not at all ( )

**13. How do you perceive your overall health?**

My health is very poor ( )

My health is poor ( )

My health is fairly poor ( )

My health is neither poor nor good ( )

My health is fairly good ( )

My health is good ( )

My health is very good ( )

**Comments on Questions 1-13.**

I Describe your work situation. (Put on X in the box that correspond best to your situation during the past week).

I work full-time ( )

I work part-time ( )

I am not gainfully employed ( )

14a. If you are gainfully employed, how happy are you with your work situation?

I am very unhappy with my work situation ( )

I am unhappy with my work situation ( )

I am fairly unhappy with my work situation ( )

I am neither unhappy nor happy with my work situation ( )

I am fairly happy with my work situation ( )

I am happy with my work situation ( )

I am very happy with my work situation ( )

**II. If you are not gainfully employed, explain why.**

I am unemployed ( )

I am a housewife ( )

I am sick-listed ( )

I am on sick pension ( )

I am an old-age pensioner ( )

I am unable to work ( )

I do not want to work because i want to do other things ( )

Other reasons ( )

**14b. If you are not gainfully employed, how happy are you with your life situation?**

I am very unhappy with my life situation ( )

I am unhappy with my life situation ( )

I am fairly unhappy with my life situation ( )

I am neither unhappy nor happy with my life situation ( )

I am fairly happy with my life situation ( )

I am happy with my life situation ( )

I am very happy with my life situation ( )

**III. Has your financial situation changed during the past year?**

Yes ( )

No ( )

**15. Describe your financial situation**

My finances are very unsatisfactory ( )

My finances are unsatisfactory ( )

My finances are fairly unsatisfactory ( )

My finances are neither unsatisfactory nor satisfactory ( )

My finances are fairly satisfactory ( )

My finances are satisfactory ( )

My finances are very satisfactory ( )

**16. Are you happy with where you live?**

I am very unhappy with where I live ( )

I am unhappy with where I live ( )

I am fairly unhappy with where I live ( )

I am neither unhappy nr happy with where I live ( )

I am fairly happy with where I live ( )

I am happy with where I live ( )

I am very happy with where I live ( )

**Comments on Questions 14-16**

**17. How active have you been during the past week?**

I have been very passive ( )

I have been passive ( )

I have been fairly passive ( )

I have been neither passive nor active ( )

I have been fairly active ( )

I have been active ( )

I have been very active ( )

**How do you feel about your activities (i.e. what you have done) during the past week?**

**To what extent have your activities been:**

**18. Fun/stimulating 19. Interesting**

Not at all ( ) Not at all ( )

Almost not at all ( ) Almost not at all ( )

To a low degree ( ) To a low degree ( )

To some degree ( ) To some degree ( )

To a fairly high degree ( ) To a fairly high degree ( )

To a high degree ( ) To a high degree ( )

To a very high degree ( ) To a very high degree ( )

**20. Creative 21. Independent**

Not at all ( ) Not at all ( )

Almost not at all ( ) Almost not at all ( )

To a low degree ( ) To a low degree ( )

To some degree ( ) To some degree ( )

To a fairly high degree ( ) To a fairly high degree ( )

To a high degree ( ) To a high degree ( )

To a very high degree ( ) To a very high degree ( )

**22. Useful 23. Meaningful**

Not at all ( ) Not at all ( )

Almost not at all ( ) Almost not at all ( )

To a low degree ( ) To a low degree ( )

To some degree ( ) To some degree ( )

To a fairly high degree ( ) To a fairly high degree ( )

To a high degree ( ) To a high degree ( )

To a very high degree ( ) To a very high degree ( )

**Comments on Questions 17-23**

**How do you experience your relationships with other people from the following perspectives?**

**Choose and assess a significant person from your family.**

**To what extent do you feel the relationship is:**

**24. Emotionally satisfying 25. Interesting/Exciting**

Not at all ( ) Not at all ( )

Almost not at all ( ) Almost not at all ( )

To a low degree ( ) To a low degree ( )

To some degree ( ) To some degree ( )

To a fairly high degree ( ) To a fairly high degree ( )

To a high degree ( ) To a high degree ( )

To a very high degree ( ) To a very high degree ( )

**26. Meaningful 27. Independent**

Not at all ( ) Not at all ( )

Almost not at all ( ) Almost not at all ( )

To a low degree ( ) To a low degree ( )

To some degree ( ) To some degree ( )

To a fairly high degree ( ) To a fairly high degree ( )

To a high degree ( ) To a high degree ( )

To a very high degree ( ) To a very high degree ( )

**28. Changeable/Richness of variation**

Not at all ( )

Almost not at all ( )

To a low degree ( )

To some degree ( )

To a fairly high degree ( )

To a high degree ( )

To a very high degree ( )

**Choose and assess a significant person from among your friends.**

**To what extent do you feel the relationship is:**

**29. Emotionally satisfying 30. Interesting/Exciting**

Not at all ( ) Not at all ( )

Almost not at all ( ) Almost not at all ( )

To a low degree ( ) To a low degree ( )

To some degree ( ) To some degree ( )

To a fairly high degree ( ) To a fairly high degree ( )

To a high degree ( ) To a high degree ( )

To a very high degree ( ) To a very high degree ( )

**31. Meaningful 32. Independent**

Not at all ( ) Not at all ( )

Almost not at all ( ) Almost not at all ( )

To a low degree ( ) To a low degree ( )

To some degree ( ) To some degree ( )

To a fairly high degree ( ) To a fairly high degree ( )

To a high degree ( ) To a high degree ( )

To a very high degree ( ) To a very high degree ( )

**33. Changeable/Richness of variation**

Not at all ( )

Almost not at all ( )

To a low degree ( )

To some degree ( )

To a fairly high degree ( )

To a high degree ( )

To a very high degree ( )

**Comments on Questions 24-33.**

**34. How do you perceive your overall quality of life**

My quality of life is very low ( )

My quality of life is low ( )

My quality of life is fairly low ( )

My quality of life is neither particularly low nor particularly high ( )

My quality of life is fairly high ( )

My quality of life is high ( )

My quality of life is very high ( )

**Comments on Question 34.**
